# Global computational mutagenesis provides a critical stability framework in protein structures

**DOI:** 10.1371/journal.pone.0189064

**Published:** 2017-12-07

**Authors:** Caitlyn L. McCafferty, Yuri V. Sergeev

**Affiliations:** Ophthalmic Genetics and Visual Function Branch, National Eye Institute, National Institutes of Health, Bethesda, Maryland, United States of America; Russian Academy of Medical Sciences, RUSSIAN FEDERATION

## Abstract

A protein’s amino acid sequence dictates the folds and final structure the macromolecule will form. We propose that by identifying critical residues in a protein’s atomic structure, we can select a critical stability framework within the protein structure essential to proper protein folding. We use global computational mutagenesis based on the unfolding mutation screen to test the effect of every possible missense mutation on the protein structure to identify the residues that cannot tolerate a substitution without causing protein misfolding. This method was tested using molecular dynamics to simulate the stability effects of mutating critical residues in proteins involved in inherited disease, such as myoglobin, p53, and the 15^th^ sushi domain of complement factor H. In addition we prove that when the critical residues are in place, other residues may be changed within the structure without a stability loss. We validate that critical residues are conserved using myoglobin to show that critical residues are the same for crystal structures of 6 different species and comparing conservation indices to critical residues in 9 eye disease-related proteins. Our studies demonstrate that by using a selection of critical elements in a protein structure we can identify a critical protein stability framework. The frame of critical residues can be used in genetic engineering to improve small molecule binding for drug studies, identify loss-of-function disease-causing missense mutations in genetics studies, and aide in identifying templates for homology modeling.

## Introduction

The interest of identifying residues most critical to a protein’s structure and function has existed for decades. By understanding those critical residues, we can better understand the folding mechanism and ways in which the structure may be modified. The approaches of these methods vary but are all based on phylogenic trees or data from limited experimental results. One such method involves a systematic study in a significant number of protein families, testing the statistical meaning of the Tree-determinant residues predicted by three different methods that represent the range of available approaches[1]. Another method using continuum electrostatics methods is used to identify destabilizing residues, and identifying functionally important residues in otherwise uncharacterized proteins[2]. However, others head warning to such approaches stating different aspects of protein function (enzymatic function classification, functional annotations in the form of key words, classes of cellular function, and conservation of binding sites) can only be reliably transferred between similar sequences to a modest degree[3].

More recently, a number of new methods using a combination of phylogenic and network analyses have emerged[4, 5]. Here, phylogenetic approaches were compared to several different network-based methods for the prediction of critical residues for protein function, and demonstrated that this method is superior to other methods previously employed. The results show that this method identifies critical residues for protein function and improves automatic sequence-based approaches and previous network-based approaches. However, for these methods there is a large reliance on Multiple Sequence Alignments (MSA) data, which has limited applications when it comes to identification of critical residues specifically due to uncertainty regarding the exact location of the active site.

While MSA has the ability to align sequences based on similarity, it is not without its limitations. A 2011 study that developed a benchmark for testing the efficacy and efficiency of MSA noted that some limitations include: locally conserved regions are less well aligned, motifs in natively disordered regions are often misaligned and finally the badly predicted or fragmentary protein sequences lead to a significant number of alignment errors[6].

When alignments are computed using protein sequences without the 3 dimensional structures, significant information regarding the protein’s secondary and tertiary structure is missed. In such circumstances, the conservation indices are based primarily on residue frequency, neglecting residue similarity. PROMALS3D[7] provides the option to use information about the secondary structure of using Protein Data Bank[8] (PDB). This method, however, has its own limitations when it comes to the availability of protein structures. There are few protein families in which more than one structure is available (about 25% of all PFAM families with a known structure)[9].

Because a protein’s structure is directly related to the protein’s function, the need to understand the role of individual residues in the folding mechanism is increasingly important. It is known that the protein sequence directs the folds and interactions that occurs between amino acids in the globular protein structure[10, 11] and mutations in the amino acid sequence lead to protein misfolding and often disease[12]. The interaction of different amino acid side chains leads to the folding of the protein into the most thermodynamically stable confirmations. As part of this process, the structure goes through a series of trial and error confirmations within the fitness landscape to identify the most thermodynamically stable structure[13].

Recently we developed the unfolding mutation screen (UMS)[14, 15], a global computational mutagenesis tool developed with the goal of evaluating the effect of any possible missense mutations on protein structure by using an unfolding propensity and displaying the data in the form of interactive heat maps. The unfolding propensity is calculated *in silico* from the free energy change between the mutant and wild type protein. This measure provides a normalized method of quantifying the effect of a mutation on protein folding with the value ranging from 0–1. Mutations that give an unfolding propensity of greater than 0.9 are said to have a severe destabilizing effect on the protein as demonstrated in a comparison with ~1,400 experimental mutations from Prothem Database[14]. These propensities can be used to identify residues that are critical to the protein’s fold. A critical residue is a residue that cannot be mutated to any other residue without having severe destabilizing effects on the protein’s structure. Critical residues are found using the foldability parameter, which evaluates the frequency of severe mutations that may occur at each location in the protein sequence. The foldability parameter shows higher risk positions for a loss of protein stability and is defined as the sum of the propensities greater than 0.9 at a given location in the sequence[14].

Analysis of this work has shown a number of trends in critical residues. Common critical residues are cysteines involved in disulfide bonding, glycines, and prolines. The critical residues are buried in the hydrophobic core of the protein or located at the protein surface and disrupt protein-protein interactions. A critical role of cysteines and glycines is well documented in human genetic eye diseases such as X-linked retinoschisis[16], age-related macula degeneration[17], Stargardt’s disease[18], and others. There is also numerous data suggesting these residues participate protein function. For example, conserved cysteine localized in functional sites of proteins[19], glycine residues are involved in inhibition of protein aggregation[20], and conserved prolines are critical for protein-protein interactions[21].

We propose that by identifying critical residues in a protein’s structure, we can select a protein stability framework that is critical to a protein’s folding. In our work, we use the UMS to calculate unfolding propensities for every possible missense mutation in a protein structure and identify these critical residues based on the foldability values. We have tested the importance of these residues by using molecular dynamics to evaluate stability and structural changes when the critical residues are both altered and kept in place. These changes were quantified using RMSD, Ramachandran plots, and distance matrices. In addition, we have shown that critical residues remain the same when calculated for proteins within a family. Finally, we have shown through direct comparisons with MSAs for 9 proteins that the critical residues are highly conserved. Critical stability framework is essential in understanding a role of genetic mutations in inherited disease.

## Results

We employed the unfolding mutation screen[14] to iterate through every possible amino acid substitution at a given location in a protein atomic structure to identify critical residues for protein folding and stability. We compared the critical residues to the highly conserved residues from multiple sequence alignments to show that if a residue is critical, it will be conserved by species over time. We used myoglobins to shows that the critical residues remain the same across species. Finally, we have proved the importance of the critical framework by generating two different structures. The first is the critical structure (CS) in which the critical residues are kept the same as in wild type protein, while the others are mutated. The other is the changed critical structure (ΔCS) where only the critical residues in the structure are changed. Comparisons of the stability of these structures demonstrate the importance of critical residues in forming a stable frame for the protein structure.

### Conservation vs. critical residues

To begin, we compared the residue conservation index from the PROMALS[22] multiple sequence alignment to the foldability of the same residue produced by UMS. Following our belief that foldability may be a suitable substitute for residue conservation, we have created a dataset of 9 eye disease-related proteins (S1–S3 Figs) to demonstrate the similarities of these two properties, as well as account for the differences. Table 1 summarizes principal characteristics of conservation indices and foldabilities to demonstrate the differences in the approaches.

**Table 1:** Comparison of the foldability and conservation index parameters.

Characteristic	Foldability	Conservation
Range	0–19	0–9 (integers only)
Method	UMS	MSA
Significance	17.1–19 Critical	9 Highly Conserved
Fundamental Theory	Thermodynamics	Statistics

In comparing the two scores, we considered differences in the ranges of the scores and the intervals in which the scores increase. The conservation index ranges from 0–9 with integer intervals. The foldability value covers the spectrum of rational numbers from 0–19. In order to create an accurate comparison of the location of the concentration of foldability values for each conservation index, the average foldability and standard deviation was calculated for each interval of the conservation index. This data was plotted for each of the proteins (S1 Fig). In addition, the Pearson’s r values ranged from 0.774 to 0.969.for the 9 proteins analyzed and the average Pearson’s r was 0.91 ± 0.057, indicating a good linear relationship between the parameters.

Next, a density plot was used for the highly conserved residues to show the distribution of foldabilities with a conservation index of 9. For the 9 proteins analyzed, the density plot had a bimodal distribution, a main peak around 18, indicating critical residues, and a smaller peak at lower foldabilities (S2 Fig). This, however, is not the case when we observe the distribution of conservation index values for the critical residues. For these density plots (S3 Fig), we observe a single large peak in the conservation index region on 8–9. The discrepancies between the distributions may be explained by the notion that structure is more conserved than sequence[23].

Our comparison of foldabilites with conservation indices shows that there is a strong agreement between the parameters. The more conserved a residue is the more likely it is to be a critical residue, while critical residues are highly conserved over time. The area in which the values differentiate can be explained by structure being more conserved that sequence, as we see over time by the different rates of change.

### Critical structure

**Fig 1:**
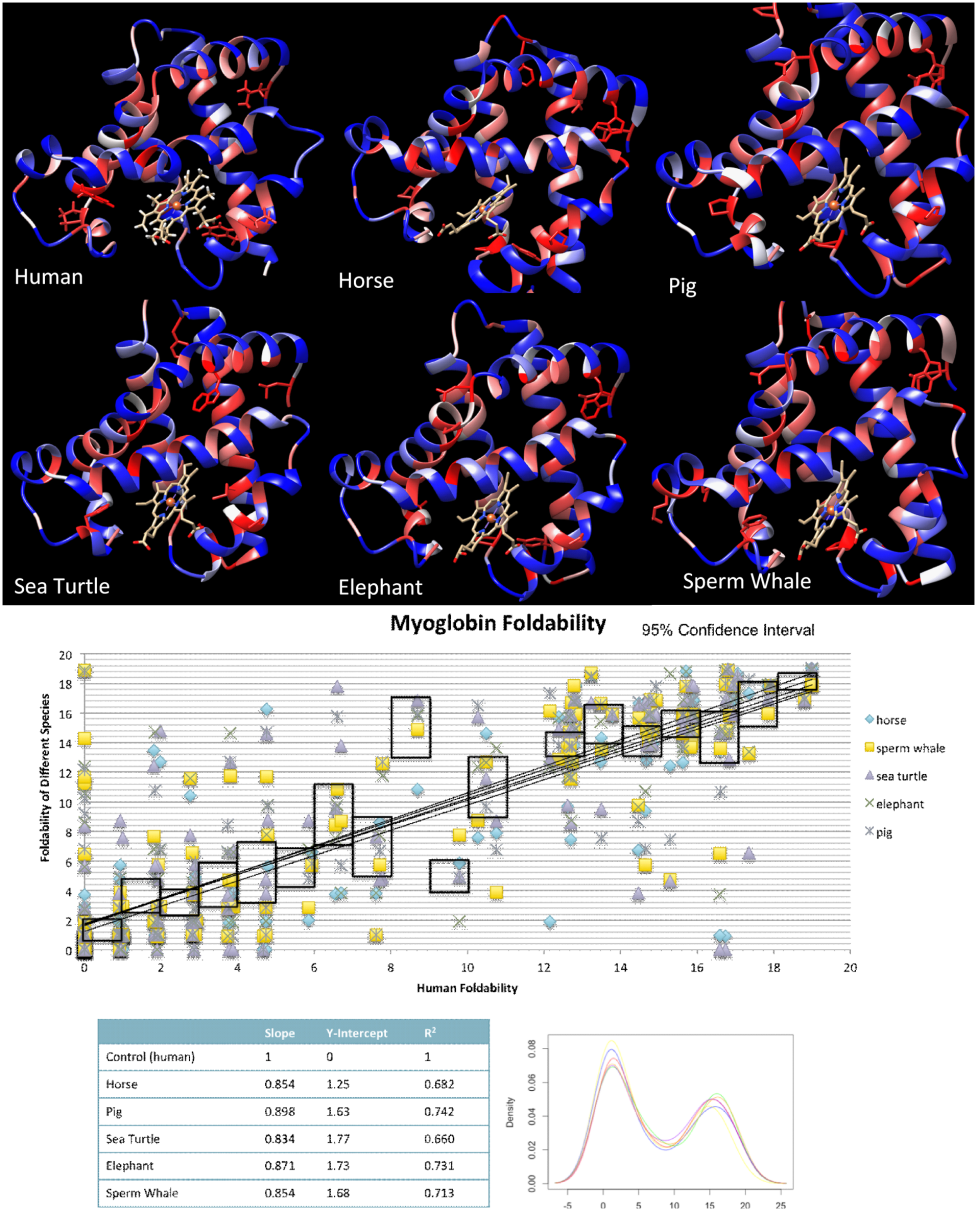
Critical residue and foldability comparison across myoglobin for 6 species. **A)** The output colored structure from UMS analysis of the 6 proteins. The red residues represent the critical residues, while the blue shows the residues that may be substituted with other residues. **B)** Pairwise comparison of human myoglobin with the 5 other species. The black outlines represent a 95% confidence interval for the data. The statistics of the graph are summarized in **C)**. **D)** The density plot shows the distribution of foldabilties in each of the structures.

Six crystal structures for myoglobin were obtained from RCSB protein databank and run through UMS to identify critical residues to ensure that critical residues were consistent among a number of species. The species used for comparison were human, horse, pig, sea turtle, elephant, and sperm whale. Fig 1a shows the patterned foldability structures side by side.

To further quantify this data, we used a pair-wise comparison method shown in Fig 1b and 1c. Human myoglobin along the x-axis serves as a benchmark, while the other species are plotted along the x-axis. The slope, y-intercept, and Pearson’s r are shown in the table. The average Pearson’s r, slope, and y-intercept for the species were 0.840 ± 0.02, 0.862 ± 0.02, and 1.61 ± 0.2, respectively. For the species, we can see some deviations between foldabilities, however, in the critical region, the values show a strong liner relationship. 95% confidence intervals are shown the black boxes to demonstrate where the majority of the values fall within the. For the critical residue region of the graph, the intervals are along the lines of best fit.

The distribution of foldabilities for each species was also plotted in a density curve (Fig 1d). The density curve shows that the distribution of foldability values is the same for each of the species. This consistency of the critical residues across species not only shows that critical residues are highly conserved, but also demonstrates that only a single atomic structure is needed to compute the critical residues for other proteins in the same family.

### Protein structure modification

**Fig 2:**
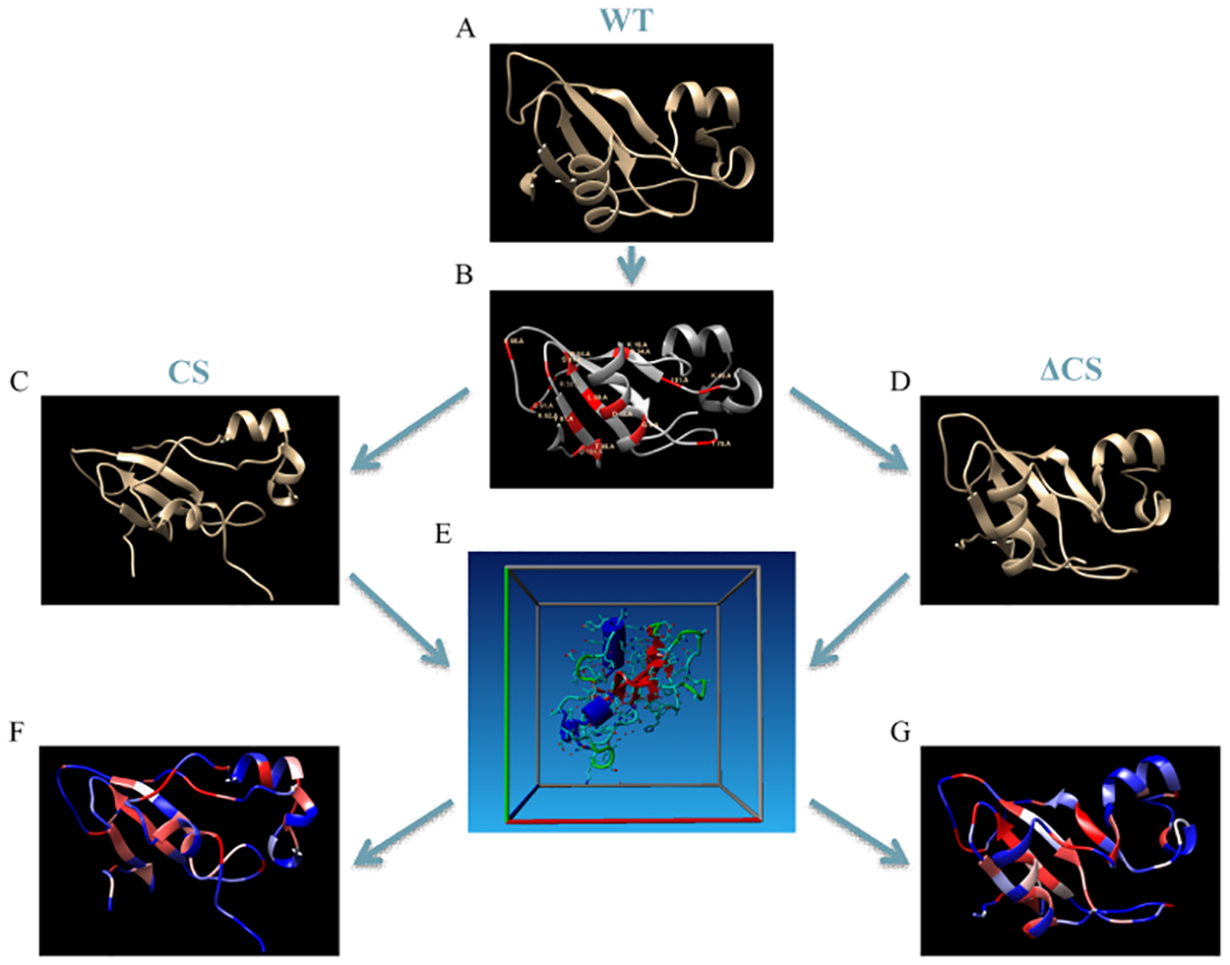
Construction and testing of the critical structure (CS) and the changed critical structure (ΔCS) proteins. **A)** The wild type protein structure is obtained from RCSB Protein Data Bank. **B)** The WT protein is run through UMS to identify the critical residues (shown in red). **C)** For the CS protein, the critical residues are kept in place and the remaining residues are mutated according to the rules of the allowed substitutions list (S4 Fig). **D)** For the ΔCS protein, each of the critical residues is mutated to alanine. **E)** Both the CS and the ΔCS structures were equilibrated in water for 100 ns as described in Methods section. **F)** The CS structure is run through UMS to identify consistencies in critical residues. **G)** The ΔCS structure is run through UMS to identify changes in critical residues.

The importance of these critical residues for preserving protein structure was tested *in silico*. Fig 2 details the computational procedure used for each of the proteins studied with this analysis. Here we have created two different structures from a single template protein. In the CS structure, the critical residues are kept in place while other residues in the structure are mutated. In the ΔCS structure, the critical residues are mutated to alanine residues.

**Fig 3:**
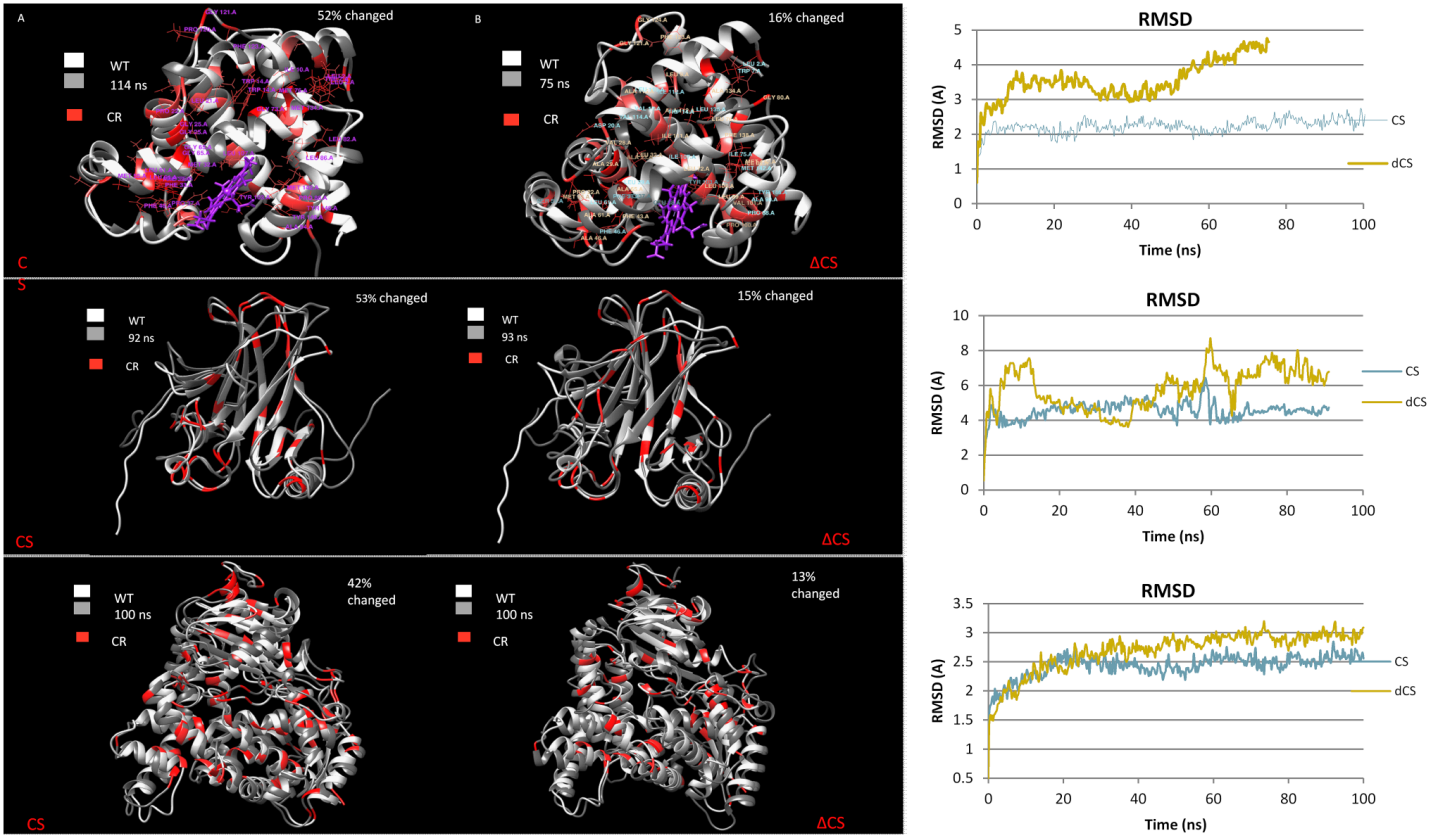
Molecular dynamics (MD) were used to simulate the affect of mutating protiens to CS and ΔCS. Critical residues for each of the structure are red and were calculated independently. **A)** 52% of noncritical human myoglobin residues were changed. The CS structure is superimposed on top of the WT human myoglobin structure. **B)** The critical residues of human myoglobin were changed to alanine residues, accounting for 12% of the residues in the structure. The ΔCS protein is superimposed on top of the WT human myoglobin structure. **C)** The RMSD for CS and ΔCS myoglobin is plotted for the MD simulation. **D)** The CS p53 with 53% of WT residues changed superimposed on the WT protein. **E)** The ΔCS p53 with 15% of residues changed superimposed on the WT protein. **F)** The RMSD for CS and ΔCS p53 is plotted for the MD simulation. **G)** The CS sushi domain 15 of complement factor H with 47% of WT residues changed superimposed on the WT protein. **H)** The ΔCS sushi domain 15 of complement factor H with 23% of residues changed superimposed on the WT protein. **I)** The RMSD for CS and ΔCS sushi domain 15 of complement factor H is plotted for the MD simulation.

Using human myoglobin, p53, and domain S15 of complement factor H as benchmarks, we tested the critical residue contribution to the protein’s structure. For the CS structures in Fig 3, the critical residues were held in place, while ~50% of the total residues were altered. The noncritical residues were mutated based on the allowed substitutions list (S4 Fig). After running the CS proteins through ~100 ns of molecular dynamics, the structures were superimposed on top of their WT structure. The RMSD of the simulation was calculated over the ~100 ns that the simulation was run (S1–S6 Tables).

The procedure was repeated but rather than maintaining the critical residues, all of the critical residues were changed to alanine (ΔCS), which accounted for 15%-23% of the total residues. The same simulation was run for ~100ns. The ΔCS structure was superimposed on the WT showing significant differences. From all three protein structures we can see that the RMSD for the ΔCS protein was larger than that for the CS protein even though much fewer of the residues were changed. The average difference between the RMSD values of the CS and ΔCS over the 100ns was as follows; domain S15 of complement factor H: 1.64Å, p53: 1.49Å, myoglobin: 1.37Å.

Following the MD simulation, both the CS proteins and the ΔCS proteins were run through UMS to identify the critical residues. The critical residues for the WT, CS, and ΔCS are shown in red on their corresponding structures (Fig 3). The superposition of these structures shows that the critical residues for the WT and CS protein align, while the critical residues for the WT and ΔCS structure do not, emphasizing the role of these critical residues in providing a critical protein stability framework.

**Fig 4:**
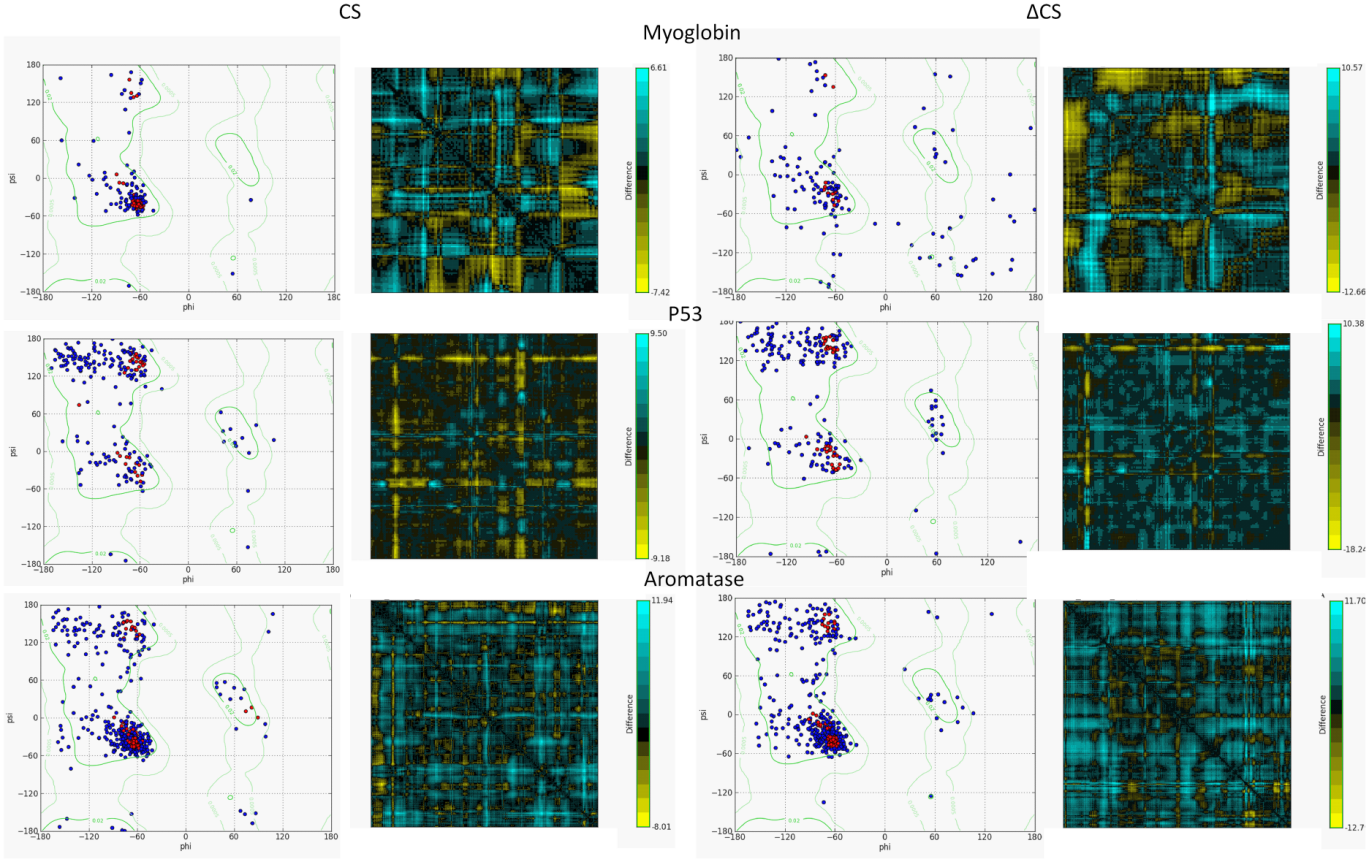
Comparison of stability between the CS and ΔCS proteins using Ramachandran plots and residue-residue distances. **A)** The plots for both the CS and ΔCS myoglobin structures. **B)** The plots for both the CS and ΔCS p53 structures. **C)** The plots for both the CS and ΔCS sushi domain 15 of complement factor H structures.

The differences between the CS and ΔCS proteins were tested using Ramachadran plots and distance maps (Fig 4). From the Ramachadran plots, it is clear that the ΔCS structure has lost much of its secondary structure and stability, while the CS structure remains stable. The distance maps show the residue-residue distance between the template protein–myoglobin, p53, and aromatase–and the modified structures (CS and ΔCS). We can see that the ΔCS structure distances are much larger than the CS. For myoglobin the distances ranged from -7.41Å to 6.61Å for the CS and -12.66 Å to 10.57Å for the ΔCS. For p53–9.18Å to 9.50Å for the CS and -18.24 Å to 10.38Å for the ΔCS. For domain S15 of complement factor H -6.05Å to 7.20Å for the CS and -17.04 Å to 24.66Å for the ΔCS.

The structure and stability differences between the CS and ΔCS show that when the critical residues are left in place, the protein can still show the proper fold even if a number of other residues in the sequence are changed. However when only the critical residues were changed there was increased stability and structure loss, despite significantly less residues being mutated. This emphasizes the importance the critical residues and their contribution to the protein’s fold.

## Discussion

**Fig 5:**
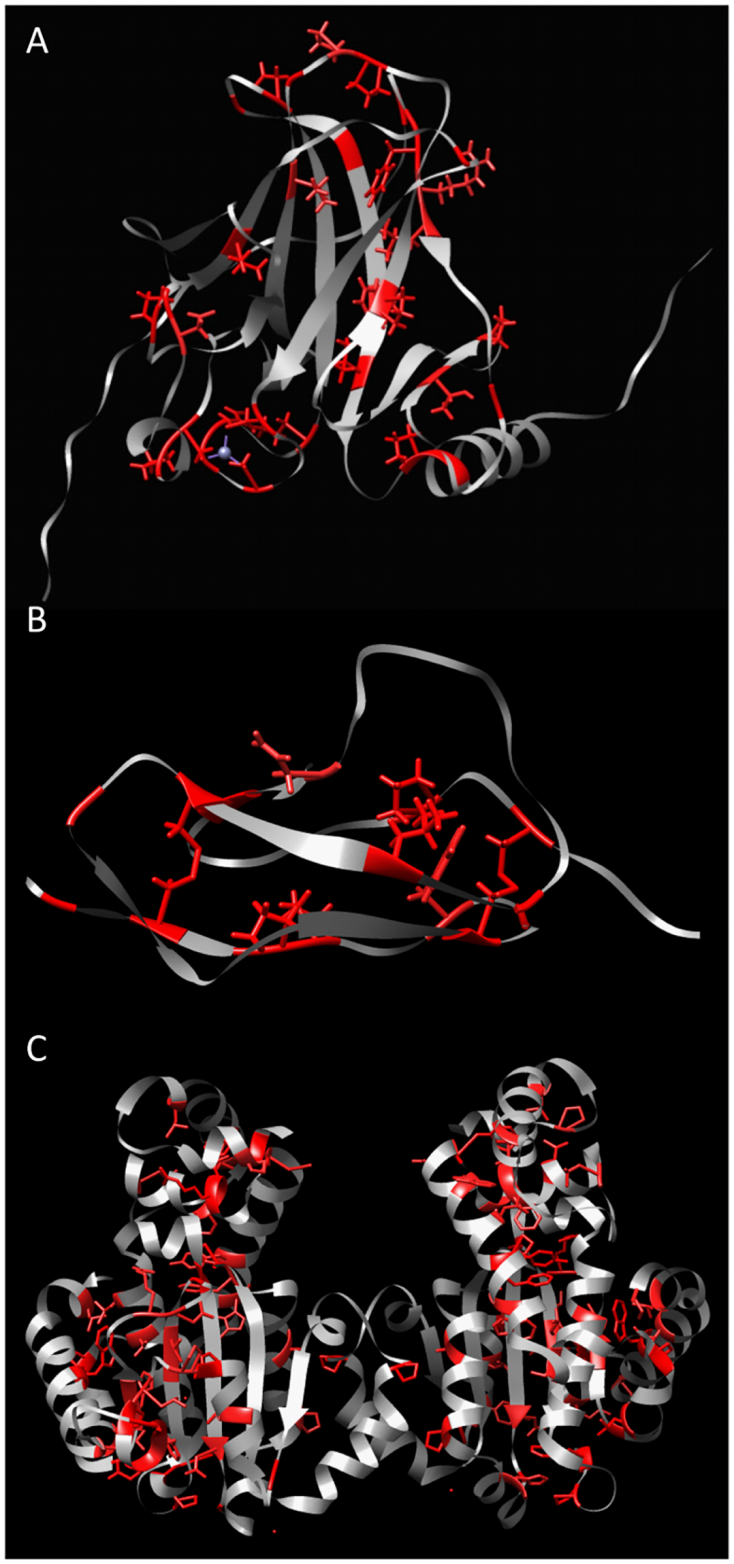
The critical residues frame for atomic protein structures. The **A)** p53, **B)** domain S15 of complement factor H, **C)** alpha-tocopherol transfer proteins are shown. The red residues represent the critical residues within the structures.

We used global computational mutagenesis to identify critical residues in protein structures. Critical residues are amino acids in the protein sequence that may not be mutated to any other amino acid without having a severe destabilizing effect of the protein structure. By iterating through every possible missense mutation in a protein sequence, we are able to isolate residues that may not be mutated to any other amino acid without leading to protein misfolding. We have then proven, *in silico*, that critical residues form a network of interactions within the protein’s structure, the critical stability framework (Fig 5). We have also shown that critical residues are highly conserved between species, further demonstrating their importance to a proteins structure.

In our studies we have examined both the critical residues’ contribution to the protein’s stability as well as the conservation of these critical residues over time. Our studies of the CS and ΔCS RMSD, Ramachandran plots, and difference contact matrices have shown that the CS remains stable supporting the stability contributions of the critical residues in creating a critical stability framework for the protein structure. Furthermore, our comparison of foldabilites with conservation indices shows that there is a strong agreement between the parameters. The more conserved a residue is the more likely it is to be a critical residue, while critical residues are highly conserved over time between species. This consistency of the critical residues across species also demonstrates that only a single atomic structure is needed to compute the critical residues for other proteins in the same family.

Protein polypeptide folds in a native protein structure within ~1 to 30 ms[24]. Unfortunately, simulations of protein folding for this range of time are computationally heavy and currently were performed just for a few proteins. In our work, the proteins were equilibrated in water for ~100 ns. In this timespan only very earlier events of protein destabilization can be modeled. However, in the 100ns the protein’s were modeled we were able to observe clear differences between CS and delta ΔCS structures to draw conclusions regarding the critical residues’ roles in creating the critical stability framework.

Molecular dynamics is used to equilibrate the altered protein structure and show some possible stability changes within the first 100 ns of simulation. These short simulations are not enough to see more significant differences, which we might expect at >30 millisecond simulation for folding/unfolding processes. But a ‘theoretically perfect’ simulation technically is not possible because of technical limitations and accuracy of current computational methods. In a future, the role of the protein stability network in protein structure can be addressed experimentally using multiple site mutagenesis. Recently, we used this method to a limited number of amino acid residues to show a role of protein glycosylation in protein stability[25]. At present, we are going to confirm the conclusions biochemically using multiple site directed mutagenesis. These experiments also could explain any significance change in trajectories for a particular molecule.

The attempt of identifying critical residues in protein structures has been implemented before[26–28], but many of these experimental techniques require a prior knowledge of the protein function and residue roles. The experimental methods involve mutating known residues involved in binding or located in active sites. The effect of these mutations is then monitored using either activity or binding. Our approach differs in its use of global computational mutagenesis based on the unfolding mutation screen. By using UMS, no prior knowledge of the protein function is required. We are able to screen the entire protein structure and evaluate any areas that may change protein stability as a result of a missense mutation.

Because the critical residue parameter has the potential to be more robust than the highly conserved residues, the critical residues should remain the same for all proteins in the family. In this study, myoglobin was used to demonstrate the robustness of the critical residue parameter amongst species. The stability network formed by the critical residues is essential to understanding the folding of the protein. The scope of this tool is further expanded because the critical residues have proven to be highly conserved among species. This means that even if a structure is not available for a protein of interest, a familial protein may be used in its place for the analysis.

In a traditional MSA, the similarity of amino acid changes is not considered, rather only the identity of changes. This means that the conservation scores are calculated based on amino acid frequencies at a certain position in the alignment. This is a problem because some changes between similar residues maybe well tolerated. It is known that there is a strong conservation of hydrophobicity in amino acid changes, excluding catalytic sites[29]. Another study considered frequencies in addition to physical chemistry and found that the method of scoring was more robust especially for functional sites given that functional sites are more conserved due to functional constraints[30]. Therefore it is important that amino acid similarities are given the appropriate weight when looking at substitutions.

In this work we are using the unfolding mutation screen to evaluate the severity of a single mutation to show an agreement of predicted values (~78%) with phenotypes from retinal disease and changes of protein stability for proteins from the ProTherm database [15]. The most severe mutations cause a protein instability, which has the potential to lead to a complete loss of protein function. These severe mutations make up the critical stability framework. The selection of critical stability framework displays a stable structure that is not affected by evolutionary changes in proteins from different species. Homologous proteins are good examples with identical core of 25–30% or higher. Proteins from different species were selected to have a similar core structures by evolution. Derived from a single protein structure, the critical stability framework could help for selection of residues maintaining protein function and stability. This is important for better understanding of proteins from different species, *de novo* protein design, and analysis of disease-causing mutations.

A knowledge of the critical stability framework can identify genetic mutations that will lead to inherited disease based on stability and function, and more specifically identify what mutations are loss of function mutations[14]. Because changing critical residue leads to loss of protein stable structure, it is likely that these mutations lead to disease. Currently, proteins showing the critical stability framework structures are included as a part into the database of 90 proteins from inherited eye disease (http://profold.nei.nih.gov).

In addition, critical core of protein could be used computationally as an alternative to overcome the score constraint, by using a MSA that creates the alignments based on protein structural files (PDB), known as multiple-structure alignment [31]. This method has shown to be more effective in computing more accurate multiple-sequence alignments, analyzing protein conformational changes, and computation of amino acid structure-sequence conservation with application to protein–protein docking prediction[24]. Using protein structure promises to be a good alternative but is limited by the lack of available proteins structures for a family of proteins.

It is known that structure is more conserved than sequence, which helps explain why critical residues are a more accurate method of describing protein stability and can be used to predict future evolutionary changes. The discrepancies between critical residues and highly conserved residues may highlight potential evolutionary changes structure is more conserved than sequence[32]. Studies have shown that a new protein fold can take millions of years to materialize in sequence space while new sequences develop in less than microseconds[33]. Structural cores are generally orders of magnitude more conserved than sequences[33]. By using UMS to identify critical residues in protein structure, a single atomic structure is required.

Possibly, that the method of determining template proteins is significantly less computationally expensive because rather than searching through sequence similarity for the entire sequence, only the critical structure is searched.

By identifying critical residues in a protein’s structure, we open a world of possibilities for modifying protein structure for improved binding. Small molecules have been used from cancer to genetic disease as a treatment[34, 35]. For enzymes this means a molecule that may rescue the enzymes activity[36]. By identifying the critical residues, we can better understand the chemistry of the protein and locate areas of the structure that may be modified to improve the binding chemistry of such small molecules. The idea of using mutagenesis to understand small molecule has already been explored[37]. As a computational technique, using critical residues can reduce the amount of time by identifying those residues that must remain in place for the protein to fold.

The idea of a network formed by these critical residues could also give insight into the process in which a protein folds. This network could be supported by the nucleation-condensation model and may serve as a stable transition state that forms before the rest of the protein fold into its native state. This topic has been extensively studied [38–40], but there are not computational tools that scan through the protein atomic structure to identify these networks. Previously, the importance of specific residues has been studied and determined to be an essential part of fast folding to decrease the number of conformations that need to be tested[41]. We plan to use this tool in the identification of protein transition states and nucleation sites *in vitro*.

The use of critical residues in the analysis of protein structures has a number of promising applications. It has shown to be an effective alternative to MSA, where the values showed agreement. Critical residues have also demonstrated that they create a critical stability framework for the protein folds, allowing other residues to be changed, a conclusion, which leads to a number of diverse applications.

## Methods

### Protein selection and alignment

The 9 eye disease-related proteins were selected based on previous studies using UMS[15]. Familial sequences for each protein were obtain from Uniprot[42] and downloaded in the Fasta format. The protein alignments were computed for the sequences using PROMALS[22], the conservation index is calculated as part of the online server. The scoring method used is based on frequencies of an amino acid at a given location and range from 0 to 9, 9 being the most conserved[43].

### Conservation index vs. foldability

As previously stated, the conservation indices (CI) were computed using PROMALS[22]. The foldabilities were calculated for the human proteins using UMS. Because the CIs range from 0 to 9 and are only integer values, they were plotted against the average foldability for the human protein for each integer value of the CI. The Pearson’s r was computed for the data.

Next the distribution of the foldabilities that were highly conserved (CI = 9) was plotted on a density plot generated using R. The distribution of the CIs for the critical residues (foldability >17.1) was then plotted on a density plot as well.

### Allowed substitutions

The allowed substitutions were calculated using a combination of experimental, computational, enzymatic, and physicochemical data. The experimental data was obtained from the Protherm Database[44]. From the database, thermodynamic data was collect for missense mutations using chemical denaturant methods[45]. The ΔΔG values collect from the database were converted to unfolding propensities. The substitutions with unfolding propensities between 0.3 and 0.6 were considered to be stable substitutions.

The computational data was based on 11 proteins from the UMS validation set[14] with a total of 34,060 missense mutations. The mutations were used to construct and 20 x 20 mutation matrix, where the middle diagonal cells of identical. Each cell contained the average unfolding propensity from the 11 proteins. Safe mutations were said to have an unfolding propensity between 0.3 and 0.6.

The enzymatic data was taken from a study that focused on amino acid exchangeability[46]. 9671 amino acid exchanges were studied; the exchangeability value was calculated from the mutant activity. The method was then tested in its ability to predict the effect of missense mutations, disease causing mutations, and model probability of fixed mutations in evolution. Stable substitutions were those who had activity greater then 50%.

The physicochemical data was extracted from the Grantham Matrix[47]. The matrix gives scores based on composition, polarity, and molecular volume. Values below 65 are considered to be conserved substitutions. All four of these parameters were considered in creating the list of allowed substitutions. A substitution was given a score of 1 for each test that was passed as stable, giving a max score of 4 and min of 0. For the experimental section data some substitutions were missing, these were given a score of 0.5 because they can be neither credited nor discredited.

The allowed substitutions had scores of 2.5 to 4.0 and are shown in Fig 1. After the allowed substitutions were determined, a properties key was created to identify the similarities in the amino acids being exchanged.

### Computational mutagenesis

Three proteins were selected to undergo the mutagenesis method–myoglobin, p53, and the 15^th^ sushi domain of complement factor H. For each of these template proteins, two new structures were created, the CS and the ΔCS structure. The CS structure uses the template protein and keeps the critical residues in place, but changes the other residues in the structure according the allowed substitution rules, resulting in a ~50% change in sequence. In the ΔCS structure, the critical residues of the template protein are changed to alanine, accounting for a ~15% change in sequence.

Both the CS and ΔCS structures were then equilibrated in water through 100 ns of molecular dynamics using a molecular-graphics, modeling, and simulation program Yasara[48], which is available at (http://www.yasara.org). For the Yasara run, we were using a standard macro ‘run_fast.mcr’ at the 2x2.5 fs simulation fast speed treating CS and Delta CS simulations in same conditions. In the Yasara macro simulations were performed at a pressure 1 bar and temperature 298K to achieve the experimentally determined water density of 0.997 g/ml. The physiological pH was 7.4. Ions were placed at the locations of the lowest/highest electrostatic potential until the cell is neutralized and the requested ion mass fraction 0.9% NaCl (153 mM) is reached. The location of the counter ions does not matter in practice, since they randomly diffuse away later (Yasara Structure manual). The AMBER14 forcefield was used with a periodic cell boundary and the cubic simulation cell of 78.19 x 78.19 x 78.19 Å. Long range electrostatics used a Particle Mesh Ewald algorithm with the 8.0 Å distance cutoff. The simulation frames were saved every 250 ps. The size of the simulation cells and number of water molecules per cell is shown in S7 Table. The resulting trajectories were analyzed using the Yasara macro md_analyzemul.mcr. The output gave a table of energies and RMSD values.

Both the CS and ΔCS structures were run through UMS after the MD simulation to identify critical residues for the modified structures to demonstrate that the critical stability network would remain the same. The same procedure was followed for the three template proteins–myoglobin, are p53, and the 15^th^ sushi domain of complement factor H.

## Supporting information

S1 Fig Comparison of conservation index (CI) and foldability for 9 eye disease-related proteins. The average foldability and standard deviation was calculated for each CI interval. R^2^ values are displayed for each of the graphs.
(TIF)

S2 Fig Density plots represent the distribution of foldabilities of highly conserved residues (CI = 9) for the 9 eye disease-related proteins. (TIF)

S3 Fig Density plots represent the distribution of conserved indices for critical residues (foldability>17.1) for the 9 eye disease related proteins. (TIF)

S4 Fig Allowed substitutions based on a combination of experimental, computational, enzymatic, and physicochemical data of missense mutations. The similarities between the residues are shown in parenthesis and are explained with the properties keys. Those with no substitutions listed did not show significant stability with any substitution.
(TIF)

S1 Table The trajectories of the simulation for human myoglobin (CS structure). (TAB)

S2 Table The trajectories of the simulation for human myoglobin (deltaCS structure). (TAB)

S3 Table The trajectories of the simulation for p53 (CS structure). (TAB)

S4 Table The trajectories of the simulation for p53 (deltaCS structure). (TAB)

S5 Table The trajectories of the simulation for domain S15 of complement factor H (CS structure). (TAB)

S6 Table The trajectories of the simulation for domain S15 of complement factor H (deltaCS structure). (TAB)

S7 Table The dimensions of the simulations cells for each of the MD simulations. The cells were cubes therefore the number indicates the length of each of the sides of the cell. The number of water molecules is shown as well.
(TIF)
