# Correction: Global computational mutagenesis provides a critical stability framework in protein structures

**DOI:** 10.1371/journal.pone.0191881

**Published:** 2018-01-24

**Authors:** Caitlyn L. McCafferty, Yuri V. Sergeev

There are several errors in Fig 1. The panels do not have labels and in the figure caption, the word “foldabilties” should read “foldabilities.” Please see the corrected Fig 1 and caption here.

**Fig 1:**
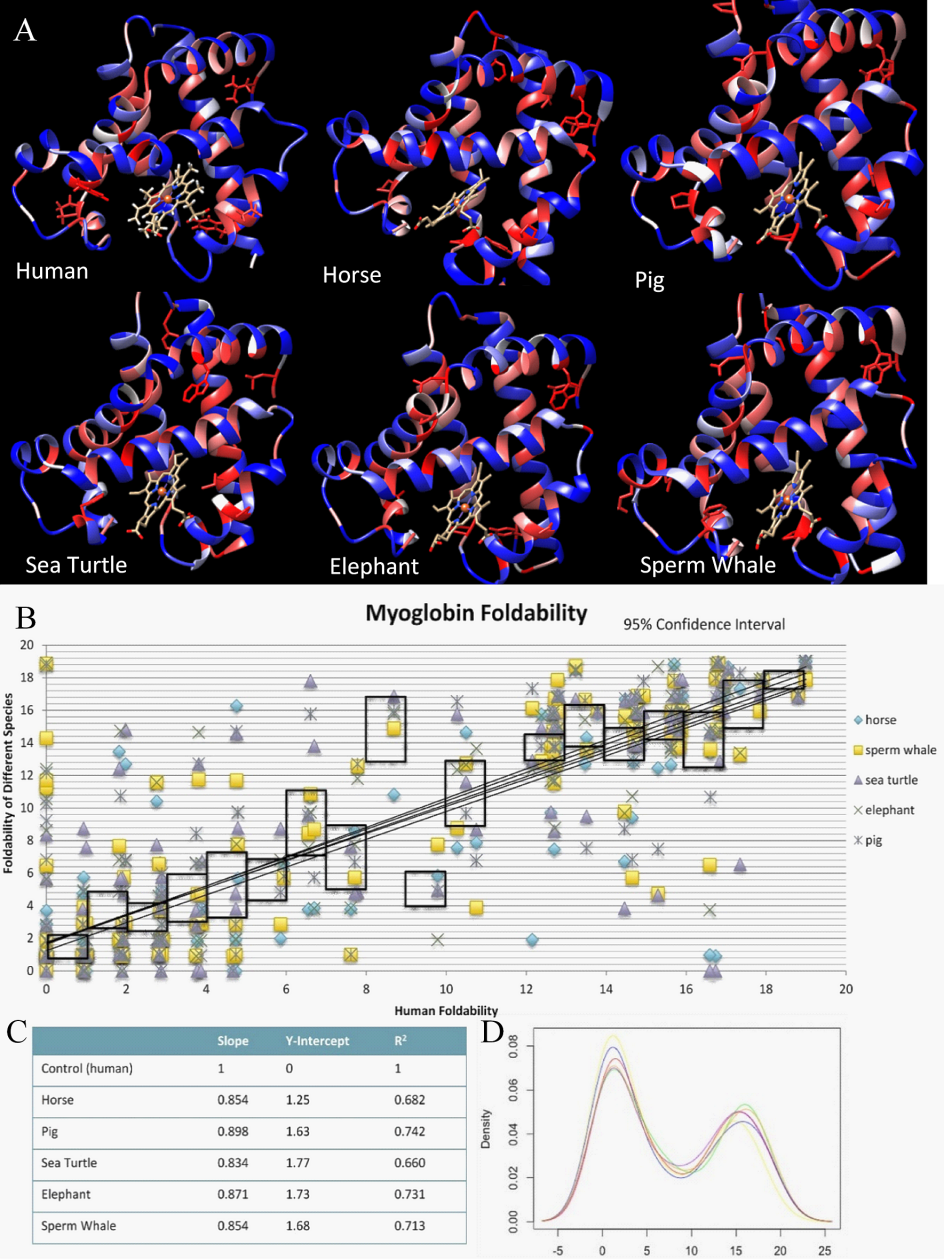
Critical residue and foldability comparison across myoglobin for 6 species. A) The output colored structure from UMS analysis of the 6 proteins. The red residues represent the critical residues, while the blue shows the residues that may be substituted with other residues. B) Pairwise comparison of human myoglobin with the 5 other species. The black outlines represent a 95% confidence interval for the data. The statistics of the graph are summarized in C). D) The density plot shows the distribution of foldabilities in each of the structures. https://doi.org/10.1371/journal.pone.0189064.g001.

There are multiple errors in Fig 3. Fig 3 shows aromatase instead of CFH15. In the caption of Fig 3, the phrase “affect of mutating protiens” should read “effect of mutating protein”. Please view the correct Fig 3 and caption here.

**Fig 3:**
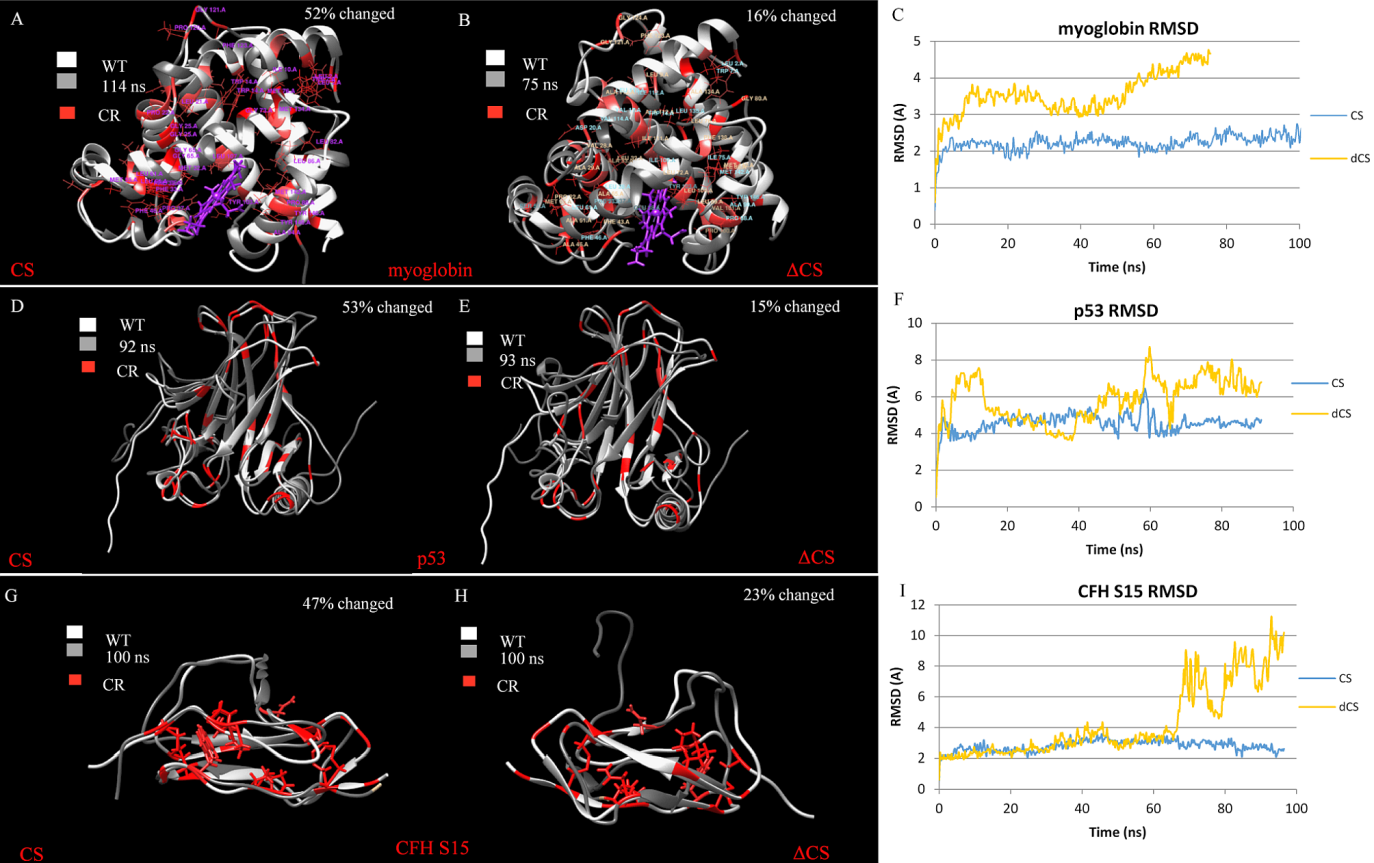
Molecular dynamics (MD) were used to simulate the effect of mutating protein to CS and ΔCS. Critical residues for each of the structure are red and were calculated independently. A) 52% of noncritical human myoglobin residues were changed. The CS structure is superimposed on top of the WT human myoglobin structure. B) The critical residues of human myoglobin were changed to alanine residues, accounting for 12% of the residues in the structure. The ΔCS protein is superimposed on top of the WT human myoglobin structure. C) The RMSD for CS and ΔCS myoglobin is plotted for the MD simulation. D) The CS p53 with 53% of WT residues changed superimposed on the WT protein. E) The ΔCS p53 with 15% of residues changed superimposed on the WT protein. F) The RMSD for CS and ΔCS p53 is plotted for the MD simulation. G) The CS sushi domain 15 of complement factor H with 47% of WT residues changed superimposed on the WT protein. H) The ΔCS sushi domain 15 of complement factor H with 23% of residues changed superimposed on the WT protein. I) The RMSD for CS and ΔCS sushi domain 15 of complement factor H is plotted for the MD simulation. https://doi.org/10.1371/journal.pone.0189064.g003.

Fig 4 is incorrect. The fig shows aromatase instead of CFH15. Please view the correct figures and captions here.

**Fig 4:**
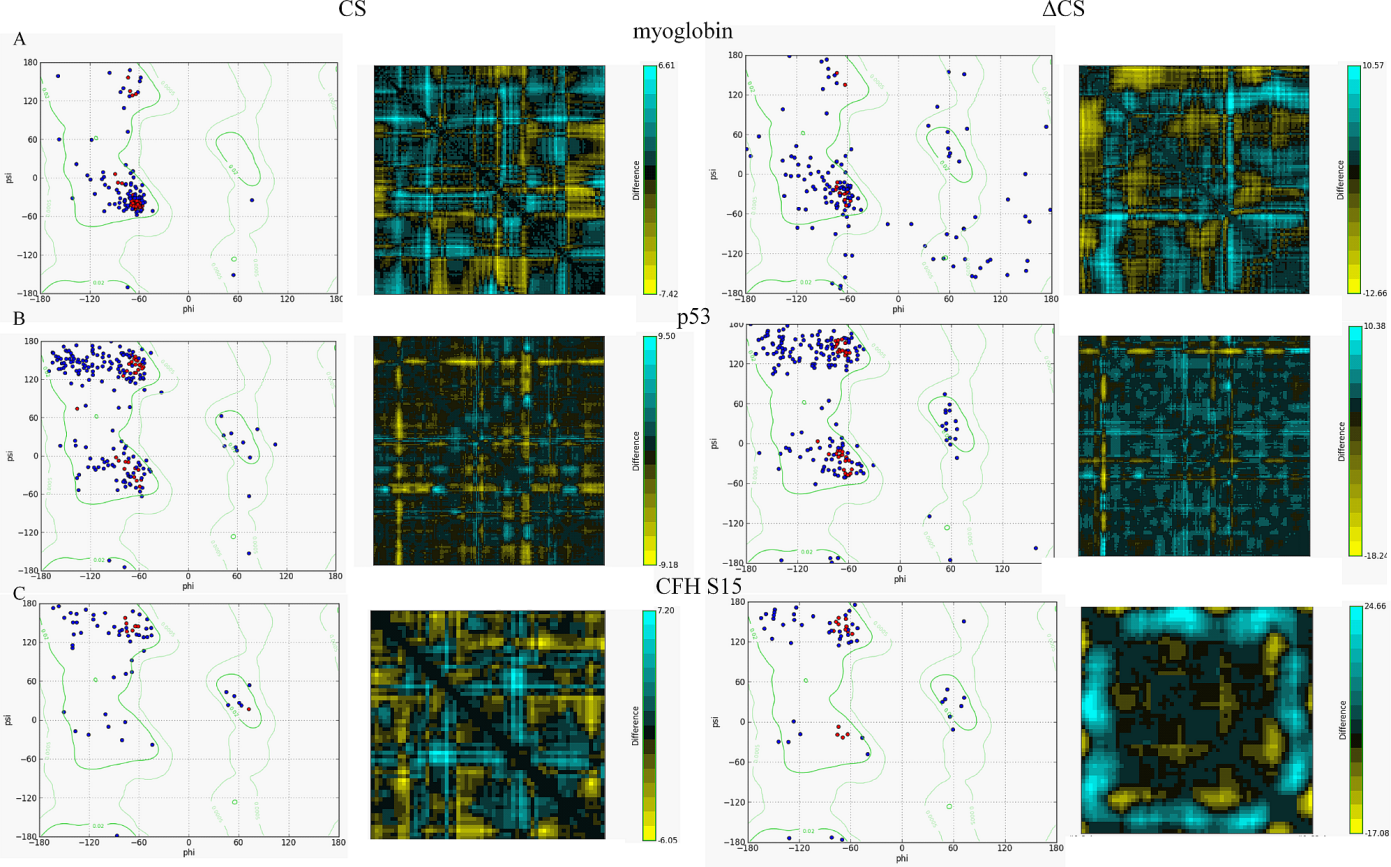
Comparison of stability between the CS and ΔCS proteins using Ramachandran plots and residue-residue distances. A) The plots for both the CS and ΔCS myoglobin structures. B) The plots for both the CS and ΔCS p53 structures. C) The plots for both the CS and ΔCS sushi domain 15 of complement factor H structures. https://doi.org/10.1371/journal.pone.0189064.g004.
