# Supplementary figures and images for: Global computational mutagenesis provides a critical stability framework in protein structures
Source: PLoS One. 2017 Dec 7;12(12):e0189064. doi: 10.1371/journal.pone.0189064 (PMC5720693; doi:10.1371/journal.pone.0189064)

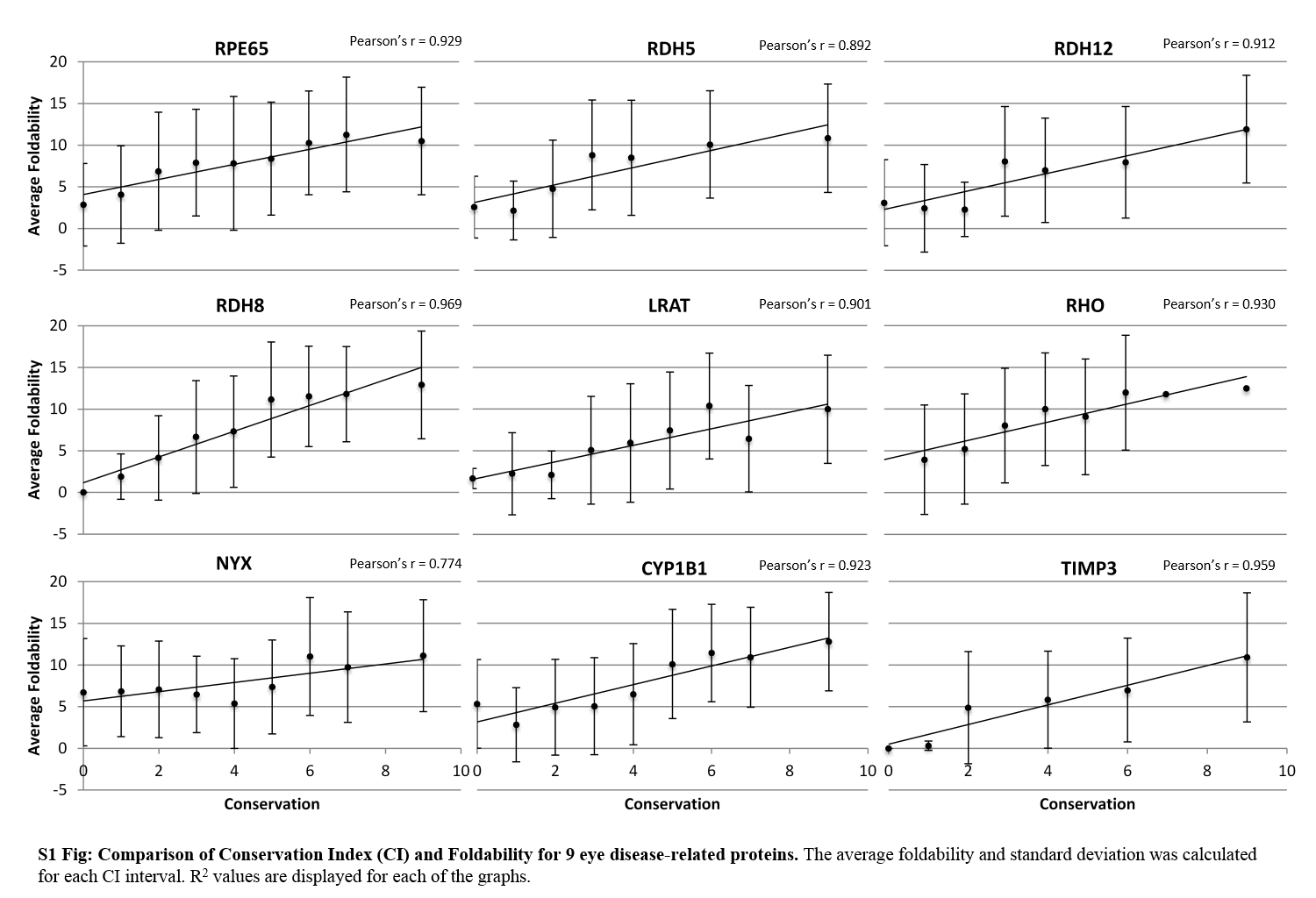

Supplement: S1 Fig — The average foldability and standard deviation was calculated for each CI interval. R2 values are displayed for each of the graphs. (TIF) [file pone.0189064.s001.tif]

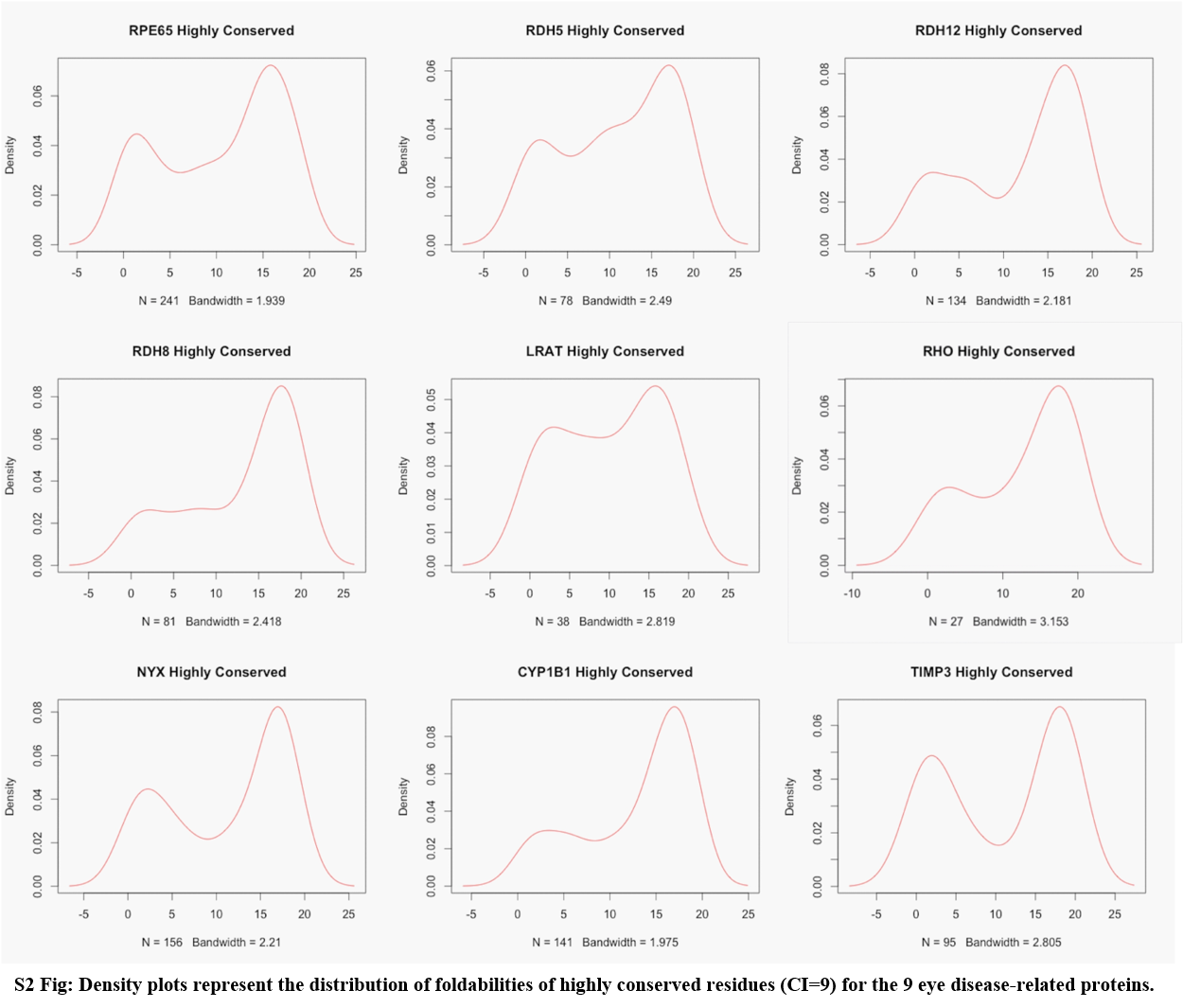

Supplement: S2 Fig — (TIF) [file pone.0189064.s002.tif]

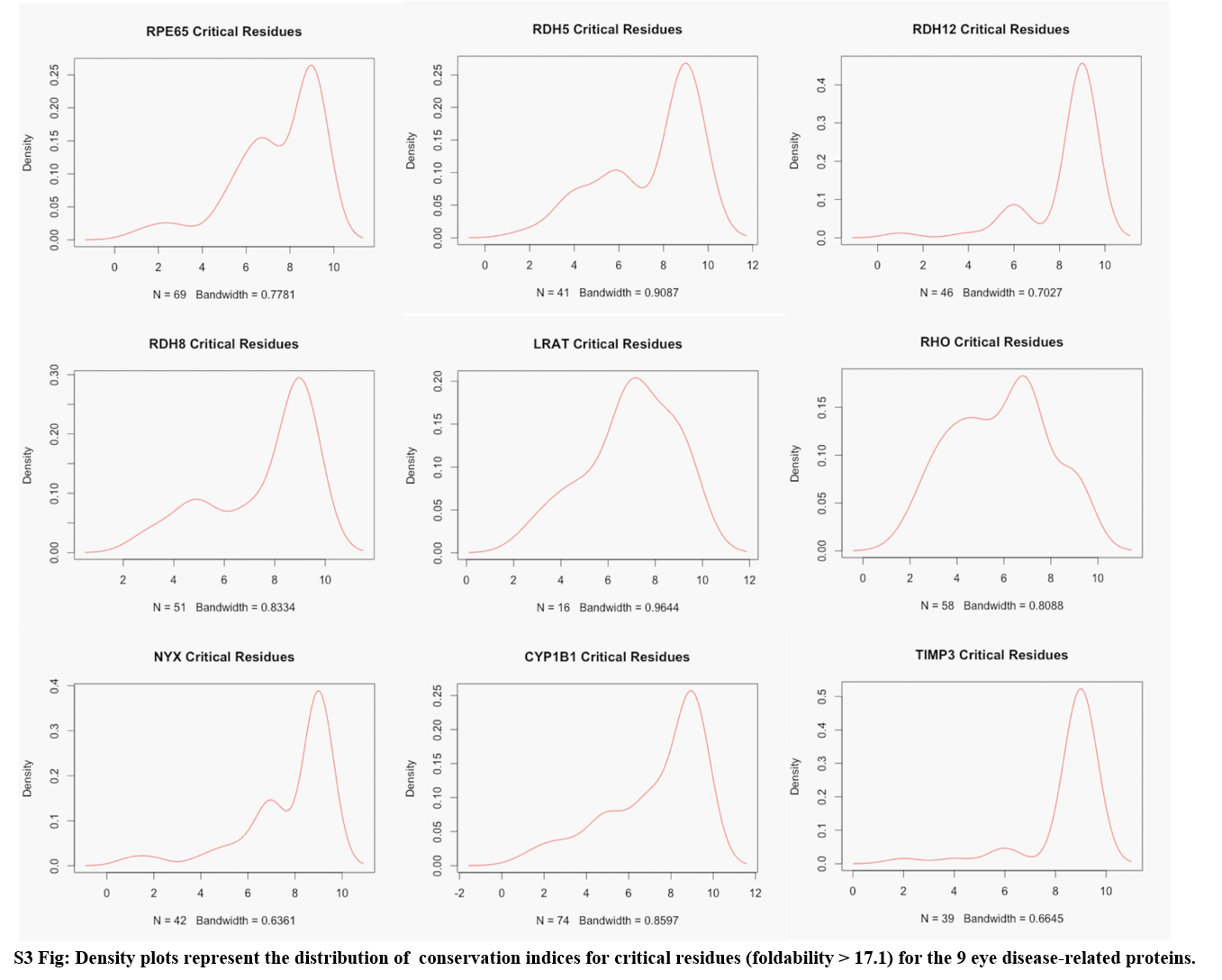

Supplement: S3 Fig — (TIF) [file pone.0189064.s003.tif]

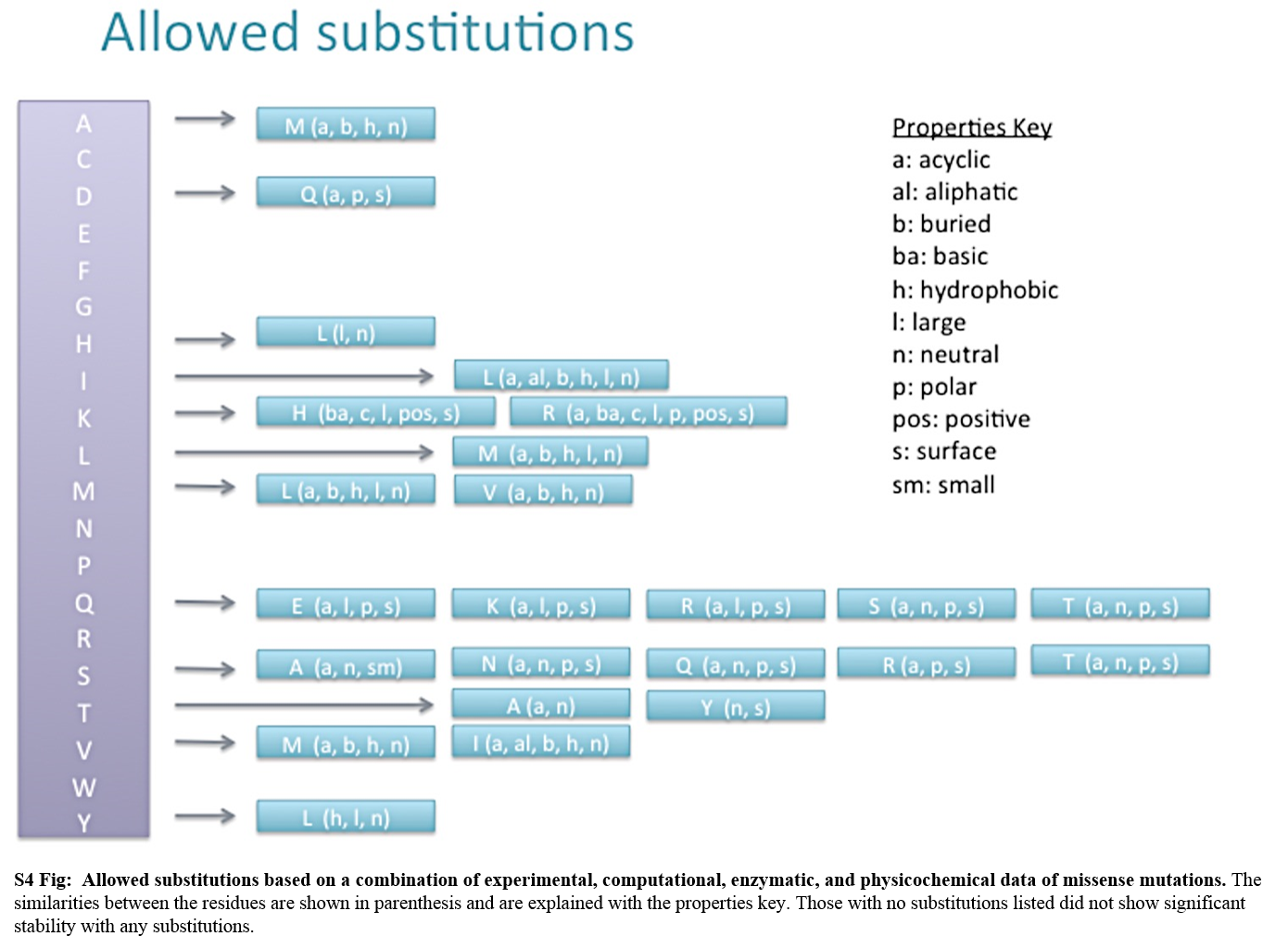

Supplement: S4 Fig — The similarities between the residues are shown in parenthesis and are explained with the properties keys. Those with no substitutions listed did not show significant stability with any substitution. (TIF) [file pone.0189064.s004.tif]

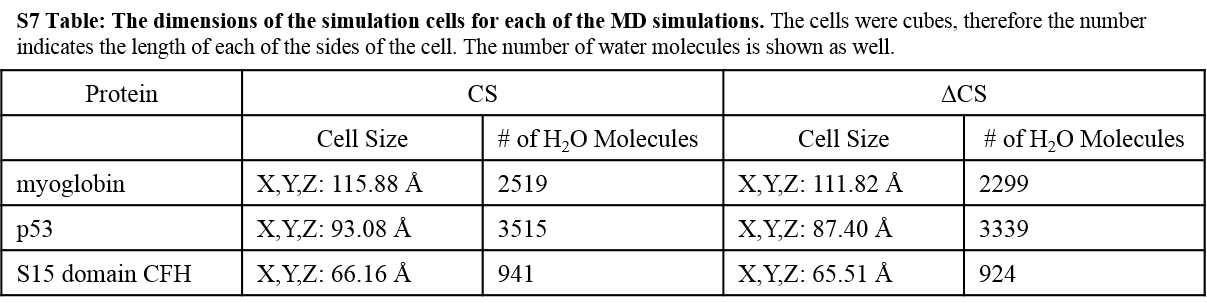

Supplement: S7 Table — The cells were cubes therefore the number indicates the length of each of the sides of the cell. The number of water molecules is shown as well. (TIF) [file pone.0189064.s011.tif]
